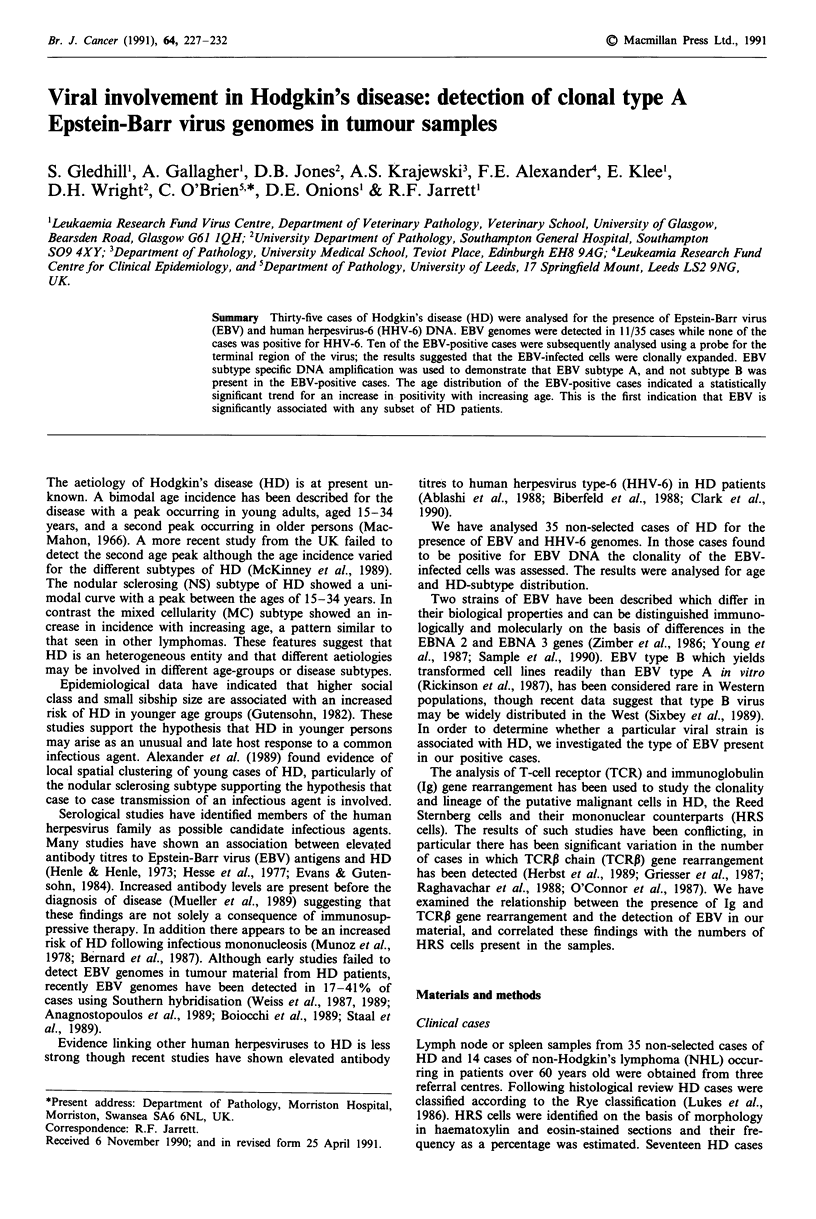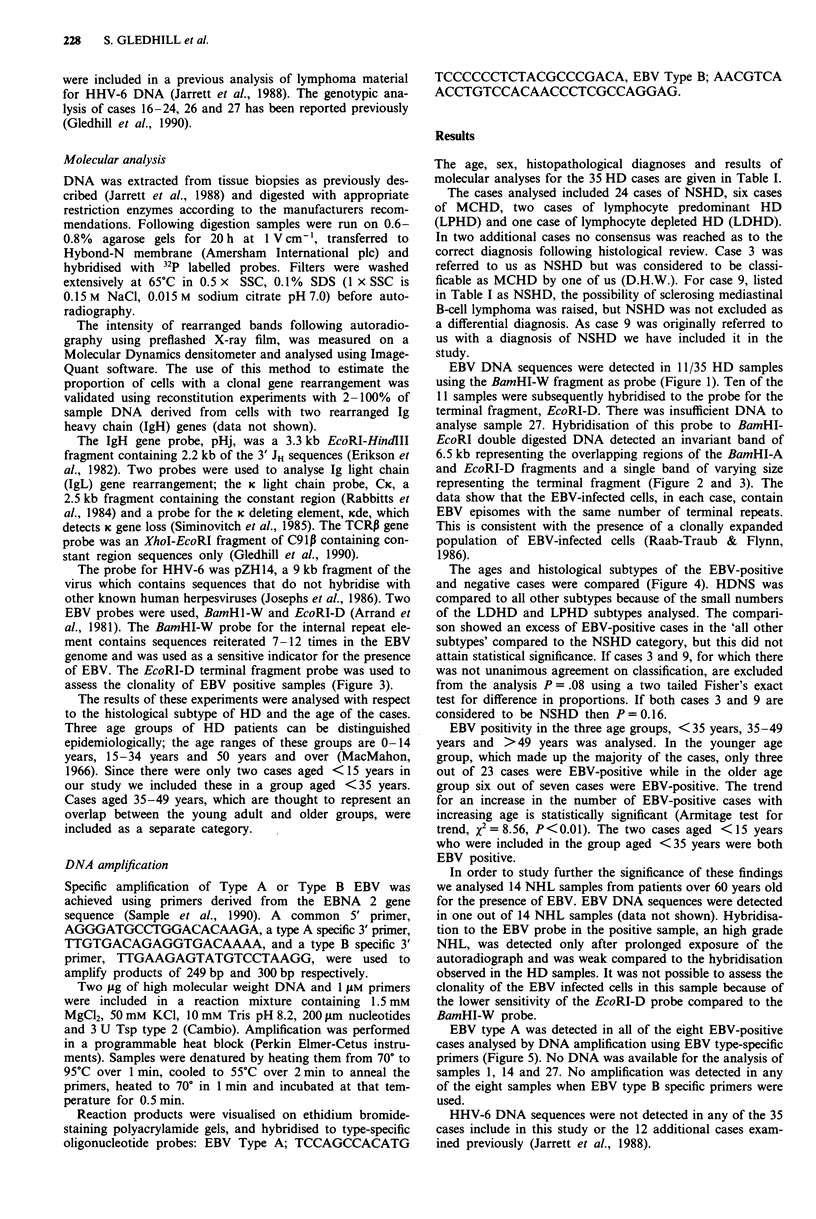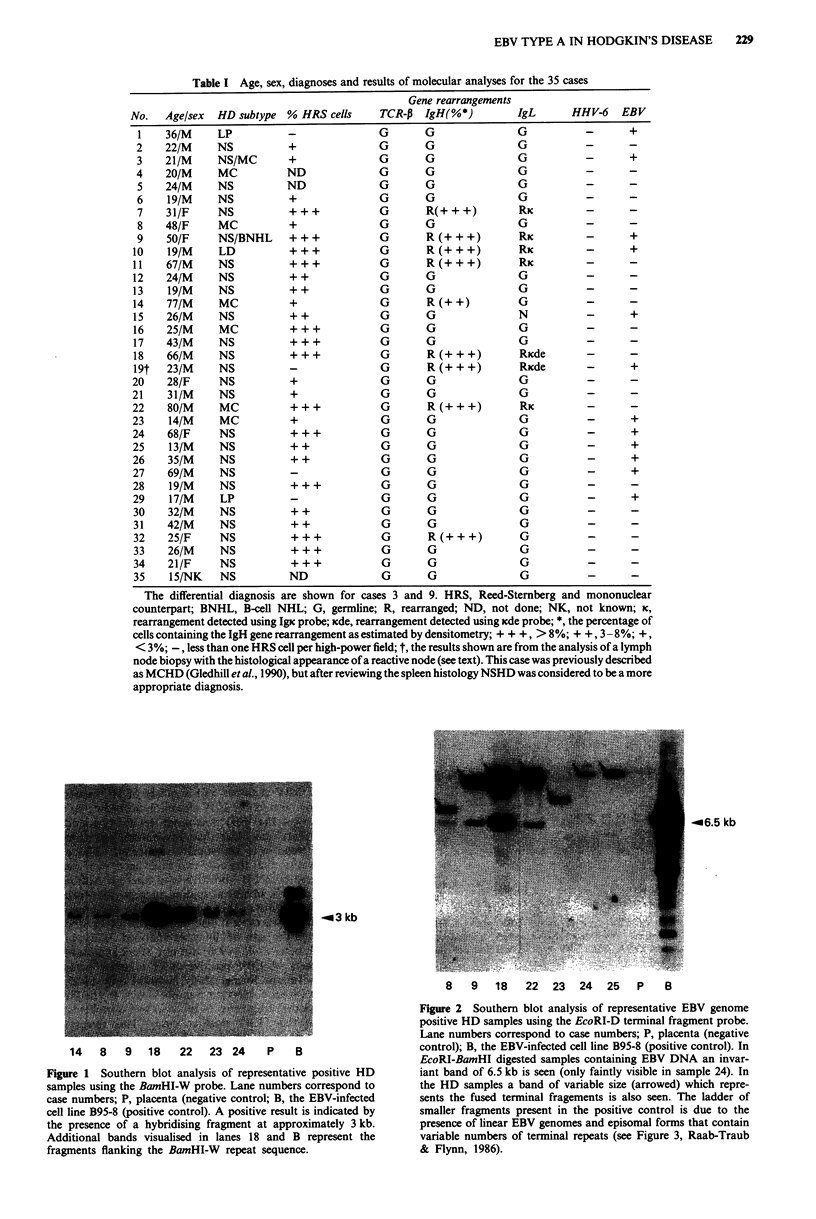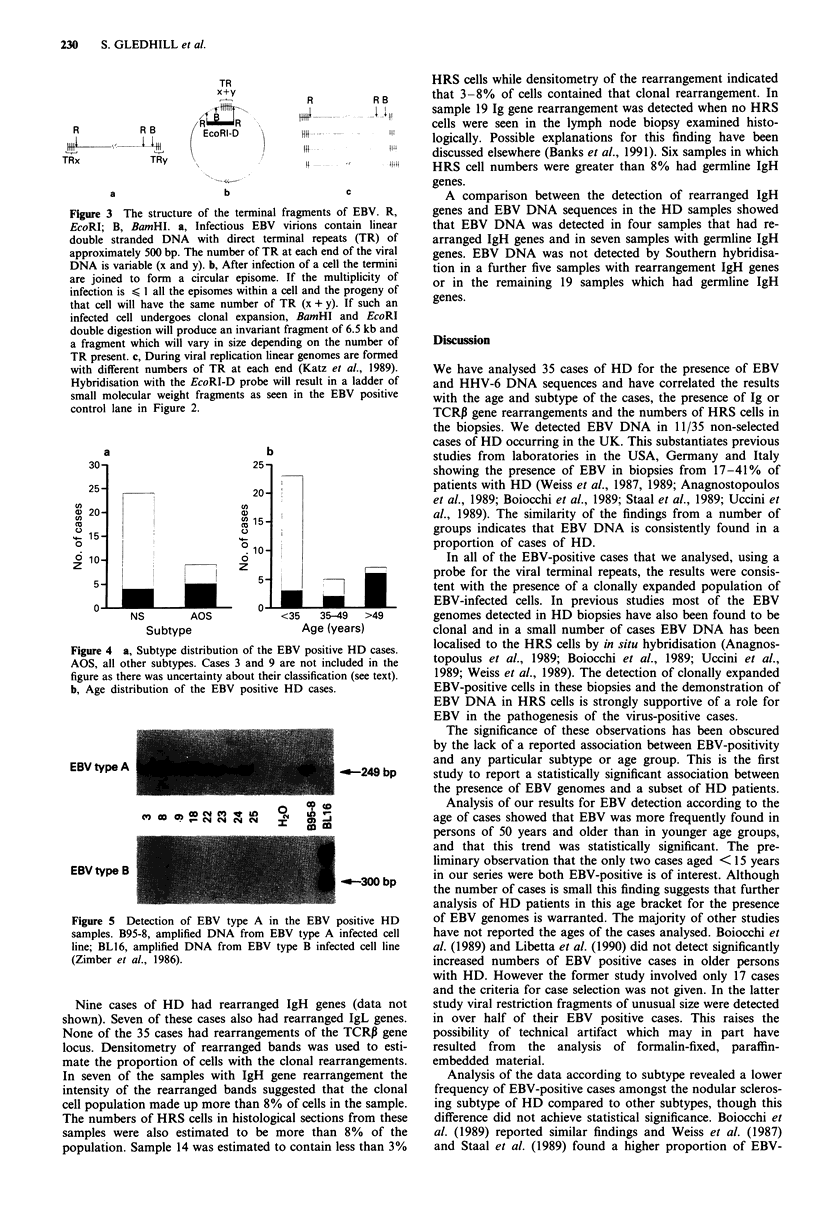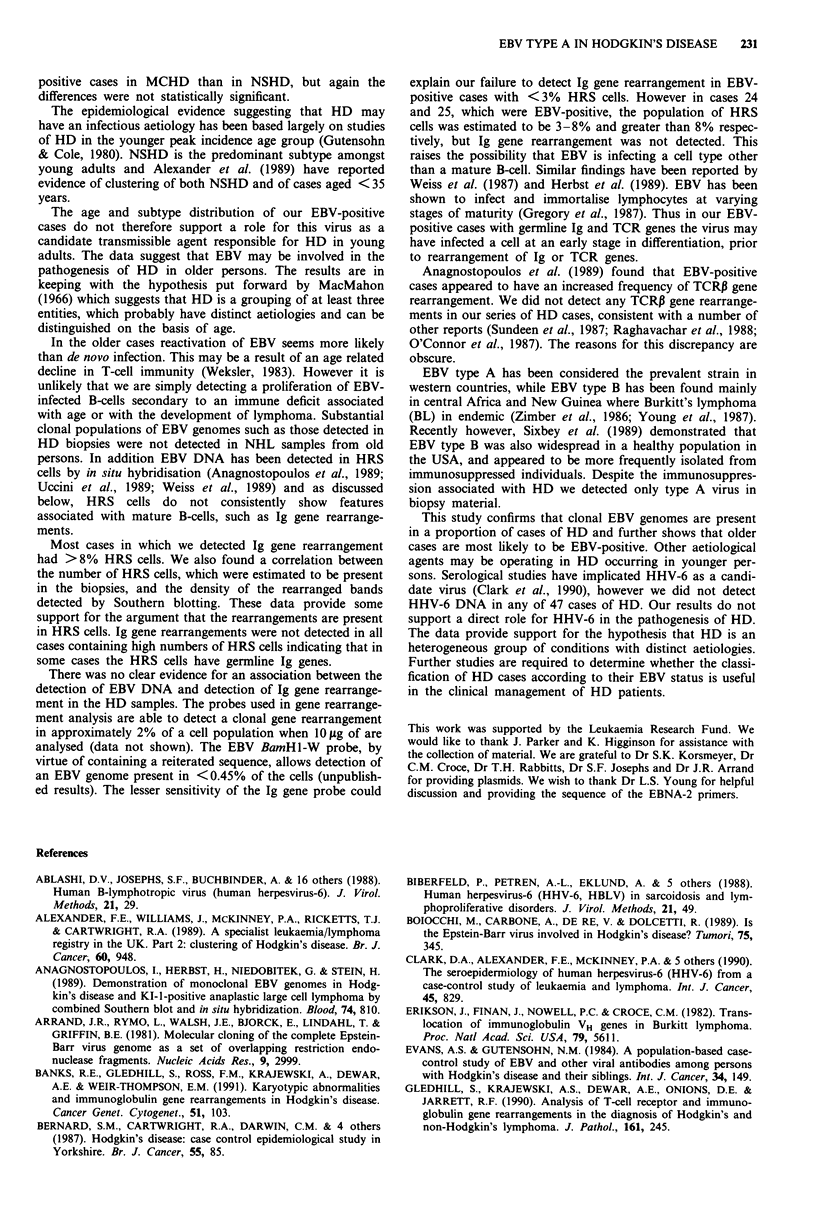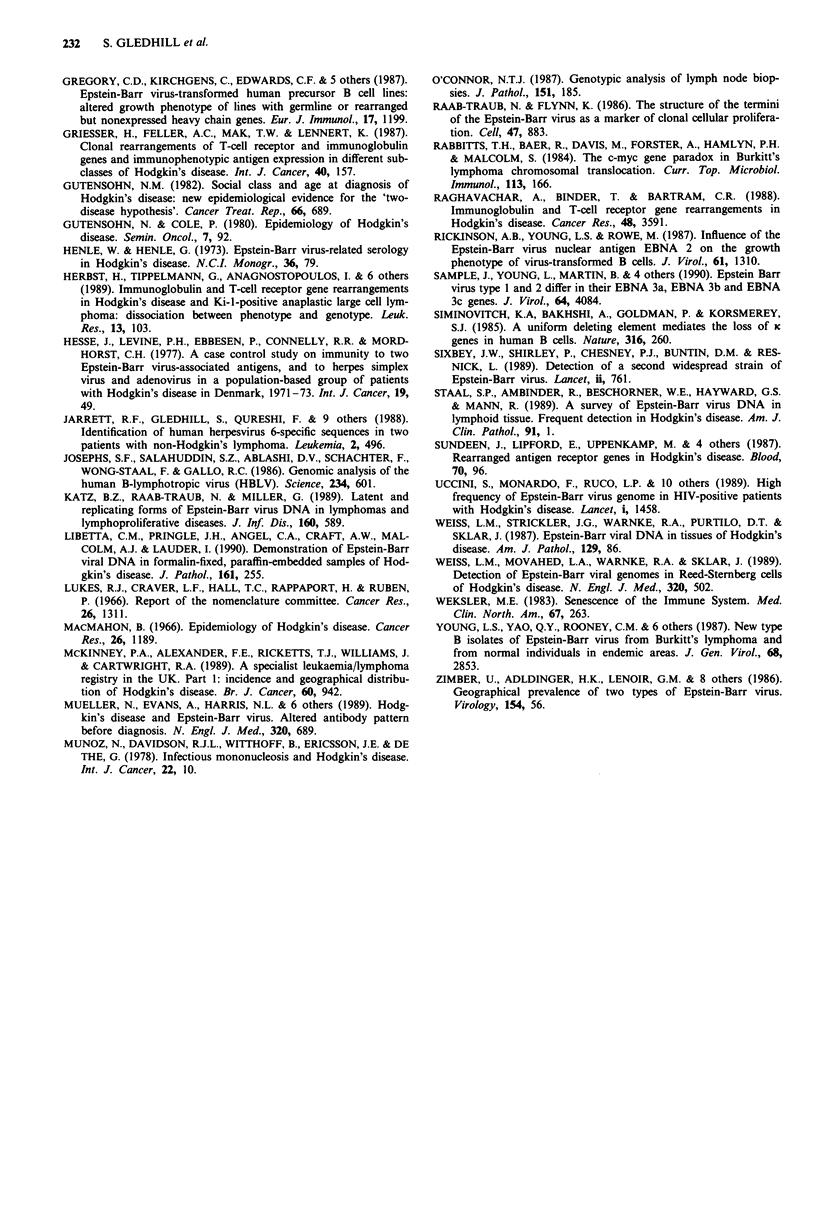# Viral involvement in Hodgkin's disease: detection of clonal type A Epstein-Barr virus genomes in tumour samples.

**DOI:** 10.1038/bjc.1991.281

**Published:** 1991-08

**Authors:** S. Gledhill, A. Gallagher, D. B. Jones, A. S. Krajewski, F. E. Alexander, E. Klee, D. H. Wright, C. O'Brien, D. E. Onions, R. F. Jarrett

**Affiliations:** Department of Veterinary Pathology, University of Glasgow, UK.

## Abstract

**Images:**


Br. J. Cancer (1991), 64, 227-232 © Macmillan Press Ltd., 1991

### Viral involvement in Hodgkin's disease: detection of clonal type A Epstein-Barr virus genomes in tumour samples

S. Gledhill[1], A. Gallagher[1], D.B. Jones[2], A.S. Krajewski[3], F.E. Alexander[4], E. Klee[1], D.H. Wright[2], C. O'Brien[5,*], D.E. Onions[1] & R.F. Jarrett[1]

[1]*Leukaemia Research Fund Virus Centre, Department of Veterinary Pathology, Veterinary School, University of Glasgow, Bearsden Road, Glasgow G61 1QH;* [2]*University Department of Pathology, Southampton General Hospital, Southampton SO9 4XY;* [3]*Department of Pathology, University Medical School, Teviot Place, Edinburgh EH8 9AG;* [4]*Leukeamia Research Fund Centre for Clinical Epidemiology, and* [5]*Department of Pathology, University of Leeds, 17 Springfield Mount, Leeds LS2 9NG, UK.*

**Summary** Thirty-five cases of Hodgkin's disease (HD) were analysed for the presence of Epstein-Barr virus (EBV) and human herpesvirus-6 (HHV-6) DNA. EBV genomes were detected in 11/35 cases while none of the cases was positive for HHV-6. Ten of the EBV-positive cases were subsequently analysed using a probe for the terminal region of the virus; the results suggested that the EBV-infected cells were clonally expanded. EBV subtype specific DNA amplification was used to demonstrate that EBV subtype A, and not subtype B was present in the EBV-positive cases. The age distribution of the EBV-positive cases indicated a statistically significant trend for an increase in positivity with increasing age. This is the first indication that EBV is significantly associated with any subset of HD patients.

The aetiology of Hodgkin's disease (HD) is at present unknown. A bimodal age incidence has been described for the disease with a peak occurring in young adults, aged 15–34 years, and a second peak occurring in older persons (MacMahon, 1966). A more recent study from the UK failed to detect the second age peak although the age incidence varied for the different subtypes of HD (McKinney *et al.*, 1989). The nodular sclerosing (NS) subtype of HD showed a unimodal curve with a peak between the ages of 15–34 years. In contrast the mixed cellularity (MC) subtype showed an increase in incidence with increasing age, a pattern similar to that seen in other lymphomas. These features suggest that HD is an heterogeneous entity and that different aetiologies may be involved in different age-groups or disease subtypes.

Epidemiological data have indicated that higher social class and small sibship size are associated with an increased risk of HD in younger age groups (Gutensohn, 1982). These studies support the hypothesis that HD in younger persons may arise as an unusual and late host response to a common infectious agent. Alexander *et al.* (1989) found evidence of local spatial clustering of young cases of HD, particularly of the nodular sclerosing subtype supporting the hypothesis that case to case transmission of an infectious agent is involved.

Serological studies have identified members of the human herpesvirus family as possible candidate infectious agents. Many studies have shown an association between elevated antibody titres to Epstein-Barr virus (EBV) antigens and HD (Henle & Henle, 1973; Hesse *et al.*, 1977; Evans & Gutensohn, 1984). Increased antibody levels are present before the diagnosis of disease (Mueller *et al.*, 1989) suggesting that these findings are not solely a consequence of immunosuppressive therapy. In addition there appears to be an increased risk of HD following infectious mononucleosis (Munoz *et al.*, 1978; Bernard *et al.*, 1987). Although early studies failed to detect EBV genomes in tumour material from HD patients, recently EBV genomes have been detected in 17–41% of cases using Southern hybridisation (Weiss *et al.*, 1987, 1989; Anagnostopoulos *et al.*, 1989; Boiocchi *et al.*, 1989; Staal *et al.*, 1989).

Evidence linking other human herpesviruses to HD is less strong though recent studies have shown elevated antibody titres to human herpesvirus type-6 (HHV-6) in HD patients (Ablashi *et al.*, 1988; Biberfeld *et al.*, 1988; Clark *et al.*, 1990).

We have analysed 35 non-selected cases of HD for the presence of EBV and HHV-6 genomes. In those cases found to be positive for EBV DNA the clonality of the EBV-infected cells was assessed. The results were analysed for age and HD-subtype distribution.

Two strains of EBV have been described which differ in their biological properties and can be distinguished immunologically and molecularly on the basis of differences in the EBNA 2 and EBNA 3 genes (Zimber *et al.*, 1986; Young *et al.*, 1987; Sample *et al.*, 1990). EBV type B which yields transformed cell lines readily than EBV type A *in vitro* (Rickinson *et al.*, 1987), has been considered rare in Western populations, though recent data suggest that type B virus may be widely distributed in the West (Sixbey *et al.*, 1989). In order to determine whether a particular viral strain is associated with HD, we investigated the type of EBV present in our positive cases.

The analysis of T-cell receptor (TCR) and immunoglobulin (Ig) gene rearrangement has been used to study the clonality and lineage of the putative malignant cells in HD, the Reed Sternberg cells and their mononuclear counterparts (HRS cells). The results of such studies have been conflicting, in particular there has been significant variation in the number of cases in which TCR$\beta$ chain (TCR$\beta$) gene rearrangement has been detected (Herbst *et al.*, 1989; Griesser *et al.*, 1987; Raghavachar *et al.*, 1988; O'Connor *et al.*, 1987). We have examined the relationship between the presence of Ig and TCR$\beta$ gene rearrangement and the detection of EBV in our material, and correlated these findings with the numbers of HRS cells present in the samples.

### Materials and methods

### *Clinical cases*

Lymph node or spleen samples from 35 non-selected cases of HD and 14 cases of non-Hodgkin's lymphoma (NHL) occurring in patients over 60 years old were obtained from three referral centres. Following histological review HD cases were classified according to the Rye classification (Lukes *et al.*, 1986). HRS cells were identified on the basis of morphology in haematoxylin and eosin-stained sections and their frequency as a percentage was estimated. Seventeen HD cases

*Present address: Department of Pathology, Morriston Hospital, Morriston, Swansea SA6 6NL, UK.
Correspondence: R.F. Jarrett.
Received 6 November 1990; and in revised form 25 April 1991.

were included in a previous analysis of lymphoma material for HHV-6 DNA (Jarrett et al., 1988). The genotypic analysis of cases 16–24, 26 and 27 has been reported previously (Gledhill et al., 1990).

*Molecular analysis*

DNA was extracted from tissue biopsies as previously described (Jarrett et al., 1988) and digested with appropriate restriction enzymes according to the manufacturers recommendations. Following digestion samples were run on 0.6–0.8% agarose gels for 20 h at 1 V $cm^{-1}$, transferred to Hybond-N membrane (Amersham International plc) and hybridised with $^{32}P$ labelled probes. Filters were washed extensively at 65°C in 0.5 × SSC, 0.1% SDS (1 × SSC is 0.15 M NaCl, 0.015 M sodium citrate pH 7.0) before autoradiography.

The intensity of rearranged bands following autoradiography using preflashed X-ray film, was measured on a Molecular Dynamics densitometer and analysed using ImageQuant software. The use of this method to estimate the proportion of cells with a clonal gene rearrangement was validated using reconstitution experiments with 2–100% of sample DNA derived from cells with two rearranged Ig heavy chain (IgH) genes (data not shown).

The IgH gene probe, pHj, was a 3.3 kb *Eco*RI-*Hind*III fragment containing 2.2 kb of the 3′ $J_H$ sequences (Erikson et al., 1982). Two probes were used to analyse Ig light chain (IgL) gene rearrangement; the κ light chain probe, Cκ, a 2.5 kb fragment containing the constant region (Rabbitts et al., 1984) and a probe for the κ deleting element, κde, which detects κ gene loss (Siminovitch et al., 1985). The TCRβ gene probe was an *Xho*I-*Eco*RI fragment of C91β containing constant region sequences only (Gledhill et al., 1990).

The probe for HHV-6 was pZH14, a 9 kb fragment of the virus which contains sequences that do not hybridise with other known human herpesviruses (Josephs et al., 1986). Two EBV probes were used, *Bam*H1-W and *Eco*RI-D (Arrand et al., 1981). The *Bam*HI-W probe for the internal repeat element contains sequences reiterated 7–12 times in the EBV genome and was used as a sensitive indicator for the presence of EBV. The *Eco*RI-D terminal fragment probe was used to assess the clonality of EBV positive samples (Figure 3).

The results of these experiments were analysed with respect to the histological subtype of HD and the age of the cases. Three age groups of HD patients can be distinguished epidemiologically; the age ranges of these groups are 0–14 years, 15–34 years and 50 years and over (MacMahon, 1966). Since there were only two cases aged <15 years in our study we included these in a group aged <35 years. Cases aged 35–49 years, which are thought to represent an overlap between the young adult and older groups, were included as a separate category.

*DNA amplification*

Specific amplification of Type A or Type B EBV was achieved using primers derived from the EBNA 2 gene sequence (Sample et al., 1990). A common 5′ primer, AGGGATGCCTGGACACAAGA, a type A specific 3′ primer, TTGTGACAGAGGTGACAAAA, and a type B specific 3′ primer, TTGAAGAGTATGTCCTAAGG, were used to amplify products of 249 bp and 300 bp respectively.

Two μg of high molecular weight DNA and 1 μM primers were included in a reaction mixture containing 1.5 mM $MgCl_2$, 50 mM KCl, 10 mM Tris pH 8.2, 200 μm nucleotides and 3 U Tsp type 2 (Cambio). Amplification was performed in a programmable heat block (Perkin Elmer-Cetus instruments). Samples were denatured by heating them from 70° to 95°C over 1 min, cooled to 55°C over 2 min to anneal the primers, heated to 70° in 1 min and incubated at that temperature for 0.5 min.

Reaction products were visualised on ethidium bromide-staining polyacrylamide gels, and hybridised to type-specific oligonucleotide probes: EBV Type A; TCCAGCCACATG TCCCCCCTCTACGCCCGACA, EBV Type B; AACGTCA ACCTGTCCACAACCCTCGCCAGGAG.

### Results

The age, sex, histopathological diagnoses and results of molecular analyses for the 35 HD cases are given in Table I.

The cases analysed included 24 cases of NSHD, six cases of MCHD, two cases of lymphocyte predominant HD (LPHD) and one case of lymphocyte depleted HD (LDHD). In two additional cases no consensus was reached as to the correct diagnosis following histological review. Case 3 was referred to us as NSHD but was considered to be classificable as MCHD by one of us (D.H.W.). For case 9, listed in Table I as NSHD, the possibility of sclerosing mediastinal B-cell lymphoma was raised, but NSHD was not excluded as a differential diagnosis. As case 9 was originally referred to us with a diagnosis of NSHD we have included it in the study.

EBV DNA sequences were detected in 11/35 HD samples using the *Bam*HI-W fragment as probe (Figure 1). Ten of the 11 samples were subsequently hybridised to the probe for the terminal fragment, *Eco*RI-D. There was insufficient DNA to analyse sample 27. Hybridisation of this probe to *Bam*HI-*Eco*RI double digested DNA detected an invariant band of 6.5 kb representing the overlapping regions of the *Bam*HI-A and *Eco*RI-D fragments and a single band of varying size representing the terminal fragment (Figure 2 and 3). The data show that the EBV-infected cells, in each case, contain EBV episomes with the same number of terminal repeats. This is consistent with the presence of a clonally expanded population of EBV-infected cells (Raab-Traub & Flynn, 1986).

The ages and histological subtypes of the EBV-positive and negative cases were compared (Figure 4). HDNS was compared to all other subtypes because of the small numbers of the LDHD and LPHD subtypes analysed. The comparison showed an excess of EBV-positive cases in the 'all other subtypes' compared to the NSHD category, but this did not attain statistical significance. If cases 3 and 9, for which there was not unanimous agreement on classification, are excluded from the analysis $P = .08$ using a two tailed Fisher's exact test for difference in proportions. If both cases 3 and 9 are considered to be NSHD then $P = 0.16$.

EBV positivity in the three age groups, <35 years, 35–49 years and >49 years was analysed. In the younger age group, which made up the majority of the cases, only three out of 23 cases were EBV-positive while in the older age group six out of seven cases were EBV-positive. The trend for an increase in the number of EBV-positive cases with increasing age is statistically significant (Armitage test for trend, $\chi^2 = 8.56$, $P < 0.01$). The two cases aged <15 years who were included in the group aged <35 years were both EBV positive.

In order to study further the significance of these findings we analysed 14 NHL samples from patients over 60 years old for the presence of EBV. EBV DNA sequences were detected in one out of 14 NHL samples (data not shown). Hybridisation to the EBV probe in the positive sample, an high grade NHL, was detected only after prolonged exposure of the autoradiograph and was weak compared to the hybridisation observed in the HD samples. It was not possible to assess the clonality of the EBV infected cells in this sample because of the lower sensitivity of the *Eco*RI-D probe compared to the *Bam*HI-W probe.

EBV type A was detected in all of the eight EBV-positive cases analysed by DNA amplification using EBV type-specific primers (Figure 5). No DNA was available for the analysis of samples 1, 14 and 27. No amplification was detected in any of the eight samples when EBV type B specific primers were used.

HHV-6 DNA sequences were not detected in any of the 35 cases include in this study or the 12 additional cases examined previously (Jarrett et al., 1988).

**Table I** Age, sex, diagnoses and results of molecular analyses for the 35 cases

| | | | | Gene rearrangements | | | | |
|---|---|---|---|---|---|---|---|---|
| *No.* | *Age/sex* | *HD subtype* | *% HRS cells* | *TCR-β* | *IgH(%*)* | *IgL* | *HHV-6* | *EBV* |
| 1 | 36/M | LP | – | G | G | G | – | + |
| 2 | 22/M | NS | + | G | G | G | – | – |
| 3 | 21/M | NS/MC | + | G | G | G | – | + |
| 4 | 20/M | MC | ND | G | G | G | – | – |
| 5 | 24/M | NS | ND | G | G | G | – | – |
| 6 | 19/M | NS | + | G | G | G | – | – |
| 7 | 31/F | NS | + + + | G | R(+ + +) | Rκ | – | – |
| 8 | 48/F | MC | + | G | G | G | – | – |
| 9 | 50/F | NS/BNHL | + + + | G | R (+ + +) | Rκ | – | + |
| 10 | 19/M | LD | + + + | G | R (+ + +) | Rκ | – | + |
| 11 | 67/M | NS | + + + | G | R (+ + +) | Rκ | – | – |
| 12 | 24/M | NS | + + | G | G | G | – | – |
| 13 | 19/M | NS | + + | G | G | G | – | – |
| 14 | 77/M | MC | + | G | R (+ +) | G | – | – |
| 15 | 26/M | NS | + + | G | G | N | – | + |
| 16 | 25/M | MC | + + + | G | G | G | – | – |
| 17 | 43/M | NS | + + + | G | G | G | – | – |
| 18 | 66/M | NS | + + + | G | R (+ + +) | Rκde | – | – |
| 19† | 23/M | NS | – | G | R (+ + +) | Rκde | – | + |
| 20 | 28/F | NS | + | G | G | G | – | – |
| 21 | 31/M | NS | + | G | G | G | – | – |
| 22 | 80/M | MC | + + + | G | R (+ + +) | Rκ | – | – |
| 23 | 14/M | MC | + | G | G | G | – | + |
| 24 | 68/F | NS | + + + | G | G | G | – | + |
| 25 | 13/M | NS | + + | G | G | G | – | + |
| 26 | 35/M | NS | + + | G | G | G | – | + |
| 27 | 69/M | NS | – | G | G | G | – | + |
| 28 | 19/M | NS | + + + | G | G | G | – | – |
| 29 | 17/M | LP | – | G | G | G | – | + |
| 30 | 32/M | NS | + + | G | G | G | – | – |
| 31 | 42/M | NS | + + | G | G | G | – | – |
| 32 | 25/F | NS | + + + | G | R (+ + +) | G | – | – |
| 33 | 26/M | NS | + + + | G | G | G | – | – |
| 34 | 21/F | NS | + + + | G | G | G | – | – |
| 35 | 15/NK | NS | ND | G | G | G | – | – |

The differential diagnosis are shown for cases 3 and 9. HRS, Reed-Sternberg and mononuclear counterpart; BNHL, B-cell NHL; G, germline; R, rearranged; ND, not done; NK, not known; κ, rearrangement detected using Igκ probe; κde, rearrangement detected using κde probe; *, the percentage of cells containing the IgH gene rearrangement as estimated by densitometry; + + +, > 8%; + +, 3–8%; +, < 3%; –, less than one HRS cell per high-power field; †, the results shown are from the analysis of a lymph node biopsy with the histological appearance of a reactive node (see text). This case was previously described as MCHD (Gledhill *et al.*, 1990), but after reviewing the spleen histology NSHD was considered to be a more appropriate diagnosis.

**Figure 1** Southern blot analysis of representative positive HD samples using the *Bam*HI-W probe. Lane numbers correspond to case numbers; P, placenta (negative control; B, the EBV-infected cell line B95-8 (positive control). A positive result is indicated by the presence of a hybridising fragment at approximately 3 kb. Additional bands visualised in lanes 18 and B represent the fragments flanking the *Bam*HI-W repeat sequence.

**Figure 2** Southern blot analysis of representative EBV genome positive HD samples using the *Eco*RI-D terminal fragment probe. Lane numbers correspond to case numbers; P, placenta (negative control); B, the EBV-infected cell line B95-8 (positive control). In *Eco*RI-*Bam*HI digested samples containing EBV DNA an invariant band of 6.5 kb is seen (only faintly visible in sample 24). In the HD samples a band of variable size (arrowed) which represents the fused terminal fragements is also seen. The ladder of smaller fragments present in the positive control is due to the presence of linear EBV genomes and episomal forms that contain variable numbers of terminal repeats (see Figure 3, Raab-Traub & Flynn, 1986).

**Figure 3** The structure of the terminal fragments of EBV. R, *Eco*RI; B, *Bam*HI. **a**, Infectious EBV virions contain linear double stranded DNA with direct terminal repeats (TR) of approximately 500 bp. The number of TR at each end of the viral DNA is variable (x and y). **b**, After infection of a cell the termini are joined to form a circular episome. If the multiplicity of infection is $\leqslant 1$ all the episomes within a cell and the progeny of that cell will have the same number of TR (x + y). If such an infected cell undergoes clonal expansion, *Bam*HI and *Eco*RI double digestion will produce an invariant fragment of 6.5 kb and a fragment which will vary in size depending on the number of TR present. **c**, During viral replication linear genomes are formed with different numbers of TR at each end (Katz *et al.*, 1989). Hybridisation with the *Eco*RI-D probe will result in a ladder of small molecular weight fragments as seen in the EBV positive control lane in Figure 2.

**Figure 4** **a**, Subtype distribution of the EBV positive HD cases. AOS, all other subtypes. Cases 3 and 9 are not included in the figure as there was uncertainty about their classification (see text). **b**, Age distribution of the EBV positive HD cases.

**Figure 5** Detection of EBV type A in the EBV positive HD samples. B95-8, amplified DNA from EBV type A infected cell line; BL16, amplified DNA from EBV type B infected cell line (Zimber *et al.*, 1986).

Nine cases of HD had rearranged IgH genes (data not shown). Seven of these cases also had rearranged IgL genes. None of the 35 cases had rearrangements of the TCR$\beta$ gene locus. Densitometry of rearranged bands was used to estimate the proportion of cells with the clonal rearrangements. In seven of the samples with IgH gene rearrangement the intensity of the rearranged bands suggested that the clonal cell population made up more than 8% of cells in the sample. The numbers of HRS cells in histological sections from these samples were also estimated to be more than 8% of the population. Sample 14 was estimated to contain less than 3% HRS cells while densitometry of the rearrangement indicated that 3–8% of cells contained that clonal rearrangement. In sample 19 Ig gene rearrangement was detected when no HRS cells were seen in the lymph node biopsy examined histologically. Possible explanations for this finding have been discussed elsewhere (Banks *et al.*, 1991). Six samples in which HRS cell numbers were greater than 8% had germline IgH genes.

A comparison between the detection of rearranged IgH genes and EBV DNA sequences in the HD samples showed that EBV DNA was detected in four samples that had rearranged IgH genes and in seven samples with germline IgH genes. EBV DNA was not detected by Southern hybridisation in a further five samples with rearrangement IgH genes or in the remaining 19 samples which had germline IgH genes.

### Discussion

We have analysed 35 cases of HD for the presence of EBV and HHV-6 DNA sequences and have correlated the results with the age and subtype of the cases, the presence of Ig or TCR$\beta$ gene rearrangements and the numbers of HRS cells in the biopsies. We detected EBV DNA in 11/35 non-selected cases of HD occurring in the UK. This substantiates previous studies from laboratories in the USA, Germany and Italy showing the presence of EBV in biopsies from 17–41% of patients with HD (Weiss *et al.*, 1987, 1989; Anagnostopoulos *et al.*, 1989; Boiocchi *et al.*, 1989; Staal *et al.*, 1989; Uccini *et al.*, 1989). The similarity of the findings from a number of groups indicates that EBV DNA is consistently found in a proportion of cases of HD.

In all of the EBV-positive cases that we analysed, using a probe for the viral terminal repeats, the results were consistent with the presence of a clonally expanded population of EBV-infected cells. In previous studies most of the EBV genomes detected in HD biopsies have also been found to be clonal and in a small number of cases EBV DNA has been localised to the HRS cells by *in situ* hybridisation (Anagnostopoulus *et al.*, 1989; Boiocchi *et al.*, 1989; Uccini *et al.*, 1989; Weiss *et al.*, 1989). The detection of clonally expanded EBV-positive cells in these biopsies and the demonstration of EBV DNA in HRS cells is strongly supportive of a role for EBV in the pathogenesis of the virus-positive cases.

The significance of these observations has been obscured by the lack of a reported association between EBV-positivity and any particular subtype or age group. This is the first study to report a statistically significant association between the presence of EBV genomes and a subset of HD patients.

Analysis of our results for EBV detection according to the age of cases showed that EBV was more frequently found in persons of 50 years and older than in younger age groups, and that this trend was statistically significant. The preliminary observation that the only two cases aged $<15$ years in our series were both EBV-positive is of interest. Although the number of cases is small this finding suggests that further analysis of HD patients in this age bracket for the presence of EBV genomes is warranted. The majority of other studies have not reported the ages of the cases analysed. Boiocchi *et al.* (1989) and Libetta *et al.* (1990) did not detect significantly increased numbers of EBV positive cases in older persons with HD. However the former study involved only 17 cases and the criteria for case selection was not given. In the latter study viral restriction fragments of unusual size were detected in over half of their EBV positive cases. This raises the possibility of technical artifact which may in part have resulted from the analysis of formalin-fixed, paraffin-embedded material.

Analysis of the data according to subtype revealed a lower frequency of EBV-positive cases amongst the nodular sclerosing subtype of HD compared to other subtypes, though this difference did not achieve statistical significance. Boiocchi *et al.* (1989) reported similar findings and Weiss *et al.* (1987) and Staal *et al.* (1989) found a higher proportion of EBV-

positive cases in MCHD than in NSHD, but again the differences were not statistically significant.

The epidemiological evidence suggesting that HD may have an infectious aetiology has been based largely on studies of HD in the younger peak incidence age group (Gutensohn & Cole, 1980). NSHD is the predominant subtype amongst young adults and Alexander *et al.* (1989) have reported evidence of clustering of both NSHD and of cases aged $<35$ years.

The age and subtype distribution of our EBV-positive cases do not therefore support a role for this virus as a candidate transmissible agent responsible for HD in young adults. The data suggest that EBV may be involved in the pathogenesis of HD in older persons. The results are in keeping with the hypothesis put forward by MacMahon (1966) which suggests that HD is a grouping of at least three entities, which probably have distinct aetiologies and can be distinguished on the basis of age.

In the older cases reactivation of EBV seems more likely than *de novo* infection. This may be a result of an age related decline in T-cell immunity (Weksler, 1983). However it is unlikely that we are simply detecting a proliferation of EBV-infected B-cells secondary to an immune deficit associated with age or with the development of lymphoma. Substantial clonal populations of EBV genomes such as those detected in HD biopsies were not detected in NHL samples from old persons. In addition EBV DNA has been detected in HRS cells by *in situ* hybridisation (Anagnostopoulos *et al.*, 1989; Uccini *et al.*, 1989; Weiss *et al.*, 1989) and as discussed below, HRS cells do not consistently show features associated with mature B-cells, such as Ig gene rearrangements.

Most cases in which we detected Ig gene rearrangement had $>8\%$ HRS cells. We also found a correlation between the number of HRS cells, which were estimated to be present in the biopsies, and the density of the rearranged bands detected by Southern blotting. These data provide some support for the argument that the rearrangements are present in HRS cells. Ig gene rearrangements were not detected in all cases containing high numbers of HRS cells indicating that in some cases the HRS cells have germline Ig genes.

There was no clear evidence for an association between the detection of EBV DNA and detection of Ig gene rearrangement in the HD samples. The probes used in gene rearrangement analysis are able to detect a clonal gene rearrangement in approximately 2% of a cell population when 10 $\mu$g of are analysed (data not shown). The EBV *Bam*H1-W probe, by virtue of containing a reiterated sequence, allows detection of an EBV genome present in $<0.45\%$ of the cells (unpublished results). The lesser sensitivity of the Ig gene probe could explain our failure to detect Ig gene rearrangement in EBV-positive cases with $<3\%$ HRS cells. However in cases 24 and 25, which were EBV-positive, the population of HRS cells was estimated to be 3–8% and greater than 8% respectively, but Ig gene rearrangement was not detected. This raises the possibility that EBV is infecting a cell type other than a mature B-cell. Similar findings have been reported by Weiss *et al.* (1987) and Herbst *et al.* (1989). EBV has been shown to infect and immortalise lymphocytes at varying stages of maturity (Gregory *et al.*, 1987). Thus in our EBV-positive cases with germline Ig and TCR genes the virus may have infected a cell at an early stage in differentiation, prior to rearrangement of Ig or TCR genes.

Anagnostopoulos *et al.* (1989) found that EBV-positive cases appeared to have an increased frequency of TCR$\beta$ gene rearrangement. We did not detect any TCR$\beta$ gene rearrangements in our series of HD cases, consistent with a number of other reports (Sundeen *et al.*, 1987; Raghavachar *et al.*, 1988; O'Connor *et al.*, 1987). The reasons for this discrepancy are obscure.

EBV type A has been considered the prevalent strain in western countries, while EBV type B has been found mainly in central Africa and New Guinea where Burkitt's lymphoma (BL) in endemic (Zimber *et al.*, 1986; Young *et al.*, 1987). Recently however, Sixbey *et al.* (1989) demonstrated that EBV type B was also widespread in a healthy population in the USA, and appeared to be more frequently isolated from immunosuppressed individuals. Despite the immunosuppression associated with HD we detected only type A virus in biopsy material.

This study confirms that clonal EBV genomes are present in a proportion of cases of HD and further shows that older cases are most likely to be EBV-positive. Other aetiological agents may be operating in HD occurring in younger persons. Serological studies have implicated HHV-6 as a candidate virus (Clark *et al.*, 1990), however we did not detect HHV-6 DNA in any of 47 cases of HD. Our results do not support a direct role for HHV-6 in the pathogenesis of HD. The data provide support for the hypothesis that HD is an heterogeneous group of conditions with distinct aetiologies. Further studies are required to determine whether the classification of HD cases according to their EBV status is useful in the clinical management of HD patients.

This work was supported by the Leukaemia Research Fund. We would like to thank J. Parker and K. Higginson for assistance with the collection of material. We are grateful to Dr S.K. Korsmeyer, Dr C.M. Croce, Dr T.H. Rabbitts, Dr S.F. Josephs and Dr J.R. Arrand for providing plasmids. We wish to thank Dr L.S. Young for helpful discussion and providing the sequence of the EBNA-2 primers.

### References

ABLASHI, D.V., JOSEPHS, S.F., BUCHBINDER, A. & 16 others (1988). Human B-lymphotropic virus (human herpesvirus-6). *J. Virol. Methods*, **21**, 29.

ALEXANDER, F.E., WILLIAMS, J., MCKINNEY, P.A., RICKETTS, T.J. & CARTWRIGHT, R.A. (1989). A specialist leukaemia/lymphoma registry in the UK. Part 2: clustering of Hodgkin's disease. *Br. J. Cancer*, **60**, 948.

ANAGNOSTOPOULOS, I., HERBST, H., NIEDOBITEK, G. & STEIN, H. (1989). Demonstration of monoclonal EBV genomes in Hodgkin's disease and KI-1-positive anaplastic large cell lymphoma by combined Southern blot and *in situ* hybridization. *Blood*, **74**, 810.

ARRAND, J.R., RYMO, L., WALSH, J.E., BJORCK, E., LINDAHL, T. & GRIFFIN, B.E. (1981). Molecular cloning of the complete Epstein-Barr virus genome as a set of overlapping restriction endonuclease fragments. *Nucleic Acids Res.*, **9**, 2999.

BANKS, R.E., GLEDHILL, S., ROSS, F.M., KRAJEWSKI, A., DEWAR, A.E. & WEIR-THOMPSON, E.M. (1991). Karyotypic abnormalities and immunoglobulin gene rearrangements in Hodgkin's disease. *Cancer Genet. Cytogenet.*, **51**, 103.

BERNARD, S.M., CARTWRIGHT, R.A., DARWIN, C.M. & 4 others (1987). Hodgkin's disease: case control epidemiological study in Yorkshire. *Br. J. Cancer*, **55**, 85.

BIBERFELD, P., PETREN, A.-L., EKLUND, A. & 5 others (1988). Human herpesvirus-6 (HHV-6, HBLV) in sarcoidosis and lymphoproliferative disorders. *J. Virol. Methods*, **21**, 49.

BOIOCCHI, M., CARBONE, A., DE RE, V. & DOLCETTI, R. (1989). Is the Epstein-Barr virus involved in Hodgkin's disease? *Tumori*, **75**, 345.

CLARK, D.A., ALEXANDER, F.E., MCKINNEY, P.A. & 5 others (1990). The seroepidermiology of human herpesvirus-6 (HHV-6) from a case-control study of leukaemia and lymphoma. *Int. J. Cancer*, **45**, 829.

ERIKSON, J., FINAN, J., NOWELL, P.C. & CROCE, C.M. (1982). Translocation of immunoglobulin $V_H$ genes in Burkitt lymphoma. *Proc. Natl Acad. Sci. USA*, **79**, 5611.

EVANS, A.S. & GUTENSOHN, N.M. (1984). A population-based case-control study of EBV and other viral antibodies among persons with Hodgkin's disease and their siblings. *Int. J. Cancer*, **34**, 149.

GLEDHILL, S., KRAJEWSKI, A.S., DEWAR, A.E., ONIONS, D.E. & JARRETT, R.F. (1990). Analysis of T-cell receptor and immunoglobulin gene rearrangements in the diagnosis of Hodgkin's and non-Hodgkin's lymphoma. *J. Pathol.*, **161**, 245.

GREGORY, C.D., KIRCHGENS, C., EDWARDS, C.F. & 5 others (1987). Epstein-Barr virus-transformed human precursor B cell lines: altered growth phenotype of lines with germline or rearranged but nonexpressed heavy chain genes. *Eur. J. Immunol.*, **17**, 1199.

GRIESSER, H., FELLER, A.C., MAK, T.W. & LENNERT, K. (1987). Clonal rearrangements of T-cell receptor and immunoglobulin genes and immunophenotypic antigen expression in different subclasses of Hodgkin's disease. *Int. J. Cancer*, **40**, 157.

GUTENSOHN, N.M. (1982). Social class and age at diagnosis of Hodgkin's disease: new epidemiological evidence for the 'two-disease hypothesis'. *Cancer Treat. Rep.*, **66**, 689.

GUTENSOHN, N. & COLE, P. (1980). Epidemiology of Hodgkin's disease. *Semin. Oncol.*, **7**, 92.

HENLE, W. & HENLE, G. (1973). Epstein-Barr virus-related serology in Hodgkin's disease. *N.C.I. Monogr.*, **36**, 79.

HERBST, H., TIPPELMANN, G., ANAGNOSTOPOULOS, I. & 6 others (1989). Immunoglobulin and T-cell receptor gene rearrangements in Hodgkin's disease and Ki-1-positive anaplastic large cell lymphoma: dissociation between phenotype and genotype. *Leuk. Res.*, **13**, 103.

HESSE, J., LEVINE, P.H., EBBESEN, P., CONNELLY, R.R. & MORDHORST, C.H. (1977). A case control study on immunity to two Epstein-Barr virus-associated antigens, and to herpes simplex virus and adenovirus in a population-based group of patients with Hodgkin's disease in Denmark, 1971–73. *Int. J. Cancer*, **19**, 49.

JARRETT, R.F., GLEDHILL, S., QURESHI, F. & 9 others (1988). Identification of human herpesvirus 6-specific sequences in two patients with non-Hodgkin's lymphoma. *Leukemia*, **2**, 496.

JOSEPHS, S.F., SALAHUDDIN, S.Z., ABLASHI, D.V., SCHACHTER, F., WONG-STAAL, F. & GALLO, R.C. (1986). Genomic analysis of the human B-lymphotropic virus (HBLV). *Science*, **234**, 601.

KATZ, B.Z., RAAB-TRAUB, N. & MILLER, G. (1989). Latent and replicating forms of Epstein-Barr virus DNA in lymphomas and lymphoproliferative diseases. *J. Inf. Dis.*, **160**, 589.

LIBETTA, C.M., PRINGLE, J.H., ANGEL, C.A., CRAFT, A.W., MALCOLM, A.J. & LAUDER, I. (1990). Demonstration of Epstein-Barr viral DNA in formalin-fixed, paraffin-embedded samples of Hodgkin's disease. *J. Pathol.*, **161**, 255.

LUKES, R.J., CRAVER, L.F., HALL, T.C., RAPPAPORT, H. & RUBEN, P. (1966). Report of the nomenclature committee. *Cancer Res.*, **26**, 1311.

MACMAHON, B. (1966). Epidemiology of Hodgkin's disease. *Cancer Res.*, **26**, 1189.

MCKINNEY, P.A., ALEXANDER, F.E., RICKETTS, T.J., WILLIAMS, J. & CARTWRIGHT, R.A. (1989). A specialist leukaemia/lymphoma registry in the UK. Part 1: incidence and geographical distribution of Hodgkin's disease. *Br. J. Cancer*, **60**, 942.

MUELLER, N., EVANS, A., HARRIS, N.L. & 6 others (1989). Hodgkin's disease and Epstein-Barr virus. Altered antibody pattern before diagnosis. *N. Engl. J. Med.*, **320**, 689.

MUNOZ, N., DAVIDSON, R.J.L., WITTHOFF, B., ERICSSON, J.E. & DE THE, G. (1978). Infectious mononucleosis and Hodgkin's disease. *Int. J. Cancer*, **22**, 10.

O'CONNOR, N.T.J. (1987). Genotypic analysis of lymph node biopsies. *J. Pathol.*, **151**, 185.

RAAB-TRAUB, N. & FLYNN, K. (1986). The structure of the termini of the Epstein-Barr virus as a marker of clonal cellular proliferation. *Cell*, **47**, 883.

RABBITTS, T.H., BAER, R., DAVIS, M., FORSTER, A., HAMLYN, P.H. & MALCOLM, S. (1984). The c-myc gene paradox in Burkitt's lymphoma chromosomal translocation. *Curr. Top. Microbiol. Immunol.*, **113**, 166.

RAGHAVACHAR, A., BINDER, T. & BARTRAM, C.R. (1988). Immunoglobulin and T-cell receptor gene rearrangements in Hodgkin's disease. *Cancer Res.*, **48**, 3591.

RICKINSON, A.B., YOUNG, L.S. & ROWE, M. (1987). Influence of the Epstein-Barr virus nuclear antigen EBNA 2 on the growth phenotype of virus-transformed B cells. *J. Virol.*, **61**, 1310.

SAMPLE, J., YOUNG, L., MARTIN, B. & 4 others (1990). Epstein Barr virus type 1 and 2 differ in their EBNA 3a, EBNA 3b and EBNA 3c genes. *J. Virol.*, **64**, 4084.

SIMINOVITCH, K.A, BAKHSHI, A., GOLDMAN, P. & KORSMEREY, S.J. (1985). A uniform deleting element mediates the loss of κ genes in human B cells. *Nature*, **316**, 260.

SIXBEY, J.W., SHIRLEY, P., CHESNEY, P.J., BUNTIN, D.M. & RESNICK, L. (1989). Detection of a second widespread strain of Epstein-Barr virus. *Lancet*, **ii**, 761.

STAAL, S.P., AMBINDER, R., BESCHORNER, W.E., HAYWARD, G.S. & MANN, R. (1989). A survey of Epstein-Barr virus DNA in lymphoid tissue. Frequent detection in Hodgkin's disease. *Am. J. Clin. Pathol.*, **91**, 1.

SUNDEEN, J., LIPFORD, E., UPPENKAMP, M. & 4 others (1987). Rearranged antigen receptor genes in Hodgkin's disease. *Blood*, **70**, 96.

UCCINI, S., MONARDO, F., RUCO, L.P. & 10 others (1989). High frequency of Epstein-Barr virus genome in HIV-positive patients with Hodgkin's disease. *Lancet*, **i**, 1458.

WEISS, L.M., STRICKLER, J.G., WARNKE, R.A., PURTILO, D.T. & SKLAR, J. (1987). Epstein-Barr viral DNA in tissues of Hodgkin's disease. *Am. J. Pathol.*, **129**, 86.

WEISS, L.M., MOVAHED, L.A., WARNKE, R.A. & SKLAR, J. (1989). Detection of Epstein-Barr viral genomes in Reed-Sternberg cells of Hodgkin's disease. *N. Engl. J. Med.*, **320**, 502.

WEKSLER, M.E. (1983). Senescence of the Immune System. *Med. Clin. North. Am.*, **67**, 263.

YOUNG, L.S., YAO, Q.Y., ROONEY, C.M. & 6 others (1987). New type B isolates of Epstein-Barr virus from Burkitt's lymphoma and from normal individuals in endemic areas. *J. Gen. Virol.*, **68**, 2853.

ZIMBER, U., ADLDINGER, H.K., LENOIR, G.M. & 8 others (1986). Geographical prevalence of two types of Epstein-Barr virus. *Virology*, **154**, 56.